# Trimethylamine N-Oxide as a Potential Biomarker for Cardiovascular Disease: Its Association with Dietary Sources of Trimethylamine N-Oxide and Microbiota

**DOI:** 10.5152/eurasianjmed.2023.23070

**Published:** 2023-12-01

**Authors:** Yasemin Karaagac

**Affiliations:** Department of Nutrition and Dietetic, İzmir Katip Çelebi University Faculty of Health Sciences, İzmir, Turkey

**Keywords:** Trimethylamine N-oxide, TMAO, cardiovascular disease, choline, carnitine, microbiota

## Abstract

Trimethylamine N-oxide (TMAO), the oxidized form of trimethylamine (TMA), was previously thought to be a waste product but is now considered an important risk factor for cardiovascular disease (CVD) and its comorbidities. Foods or supplements containing choline and carnitine are major precursors of TMA in the diet and are metabolized by gut microbiota. Trimethylamine N-oxide is produced through the oxidation of this compound by flavin-containing monooxygenase (FMO) in the liver. The organ responsible for the removal of TMAO from body fluids is the kidneys. Therefore, plasma TMAO levels are influenced by multiple complex factors, especially the amount of TMA precursors and dietary TMAO sources in the diet, the dominant genera in the gut microbiota, FMO3 enzyme activity, and kidney functions. Among these, the quantity of TMAO and its precursors in the diet and microbiota can be considered modifiable risk factors. However, discussions continue regarding how plasma TMAO levels reach pathological levels and their role (consequence or cause) in CVD. This review presents the current scientific evidence on the relationship and underlying mechanisms between CVD and TMAO and provides an overview of the association of plasma TMAO levels with modifiable risk factors, such as dietary TMAO precursors, dietary TMAO sources, and microbiota.

## Introduction

Trimethylamine N-oxide (TMAO), the oxidation product of trimethylamine (TMA), is an important metabolite that actively participates in various biological reactions and affects the activities of enzymes and hormones in the human body.^1^ In a study in which TMA production from dietary precursors could be observed using an in vitro human intestinal model, the conversion rate of precursors to TMA was determined, from highest to lowest, as choline, carnitine, betaine, and γ-butyrobetaine, respectively. The gut microbiota metabolizes these nutrients (especially choline and carnitine) and produces TMA.^2-6^ Trimethylamine travels via the portal circulation to the liver, where it is metabolized by flavin-containing monooxygenases (FMOs). There are 5 functional FMO isoforms (FMO1-5) in humans. Among these, the primary isoform converting TMA to TMAO is FMAO-3, and other FMO isoforms are not thought to play a substantial role in TMA oxidation in humans.^7^ Trimethylamine N-oxide is excreted from the body renally. A very large (>95%) portion of TMAO is produced in the liver, and taken from some food sources, it is excreted by the kidneys within 24 hour.^8^ Thus, kidney function serves as the key determinant of TMAO circulation levels.

**Figure 1.**
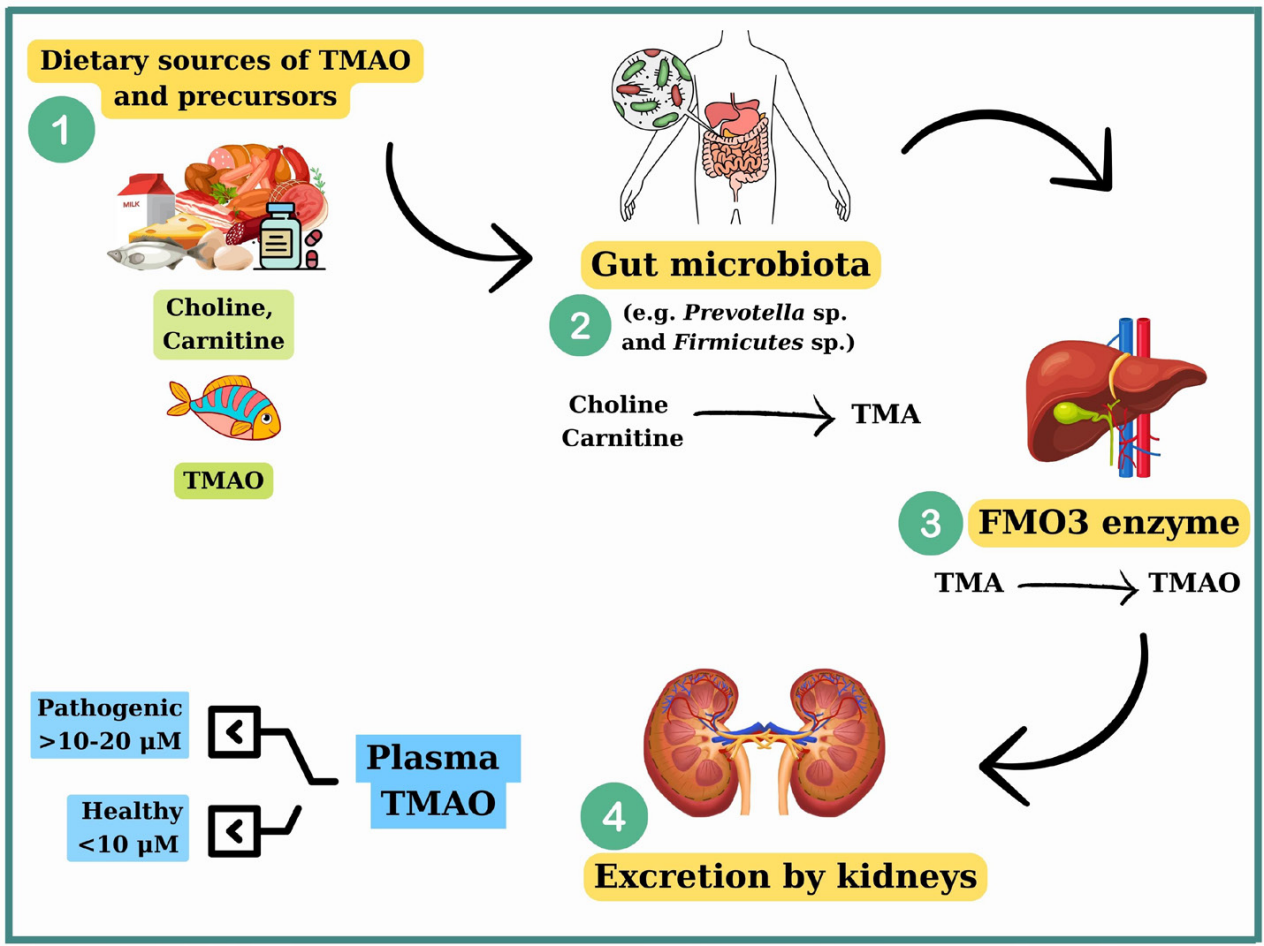
The TMAO Pathway: From Dietary Sources to Plasma Levels.

Cardiovascular disease (CVD) causes the deaths of approximately 17.9 million people each year.^9^ While it is debated whether elevated TMAO levels represent a cause or a consequence of CVD,^2-4^ elevated TMAO levels are currently an important risk factor associated with increased CVD risk, adverse cardiovascular events, and cardiovascular death.^10-18^ Plasma TMAO levels in healthy people generally range from 2 to 5 µM.^19-24^ It was stated that the plasma TMAO levels where pathogenic effects were observed are 10-20 µM/L and above.^3^ Figure 1 shows TMAO production from dietary sources of TMAO or TMAO precursors. It is acknowledged that plasma TMAO levels are influenced by multiple factors, most notably dietary intake,^25,26^ gut microbiota,^7,19,27^ FMO enzyme activity,^28,29^ and renal function.^8,20,30^ However, the regulation of circulating TMAO levels and the mechanisms that cause them to reach pathological levels have not been fully comprehended. In addition, it remains uncertain whether elevated plasma levels of TMAO are a cause or a consequence of the pathogenesis of CVD. Therefore, the relationship between plasma TMAO levels and the risk of CVD, as well as the factors associated with these levels, has become a significant area of interest in both clinical and academic circles. This review presents the current scientific evidence on the relationship and underlying mechanisms between CVD and TMAO and provides an overview of the association of plasma TMAO levels with modifiable risk factors, such as dietary TMAO precursors, dietary TMAO sources, and microbiota.

## Association Between Trimethylamine N-oxide Levels and CVD

It is now widely accepted that plasma TMAO levels predict the risk of CVD, and TMAO levels have been proposed as a biomarker for assessing CVD risk. In the meta-analysis study of Schiattarella et al,^16^ it was determined that high plasma TMAO levels increased the risk of major adverse cardiovascular events (MACEs)/cerebrovascular events by 67% and all-cause mortality by 91%. Moreover, every 10 µmol/L increase in the plasma TMAO concentration was associated with a 7.6% increase in the risk of all-cause mortality.^16^ A further meta-analysis indicated that high plasma TMAO concentrations in chronic heart patients increased the risk of MACEs by 58%, and this increase was higher (96%) in patients with longer follow-up (≥4 years). The study also indicated the existence of a J-type correlation between TMAO concentration and MACE incidence, with a TMAO level of 5.1 µmol/L potentially serving as a threshold for MACE risk in these patients.^10^ Moreover, there is evidence to suggest that elevated plasma TMAO levels are associated with an increased risk of adverse cardiovascular events by 3.3-fold in patients who have had their first ischemic stroke.^31^ High TMAO levels have also been reported to be associated with an increased risk of all-cause mortality^20,30,32^ and cardiovascular events^20,33^ in patients with chronic renal failure.

Most of the knowledge on the mechanisms by which the correlation between plasma TMAO levels and CVD risk has been established is based on cell and animal studies. In these studies, it was reported that high TMAO levels trigger^34-36^ and exacerbate^37^ vascular inflammation with proinflammatory effects, cause endothelial damage,^34^ inhibit reverse cholesterol transport,^19^ and increase the transformation of macrophages into foam cells^38^ as well as platelet activation and thrombosis formation.^39^ Some studies conducted with humans have also supported these mechanisms. For example, Chou et al (2019) determined that plasma TMAO levels were positively associated with inflammatory biomarkers, such as hs-CRP and IL-1β, and negatively associated with endothelial function markers, such as the endothelial progenitor cell count and flow-mediated vasodilation, in patients with stable angina.^36^ Haghikia et al (2018) also reported a strong positive correlation between plasma TMAO levels and the percentage of proinflammatory-mediated monocytes in patients with ischemic stroke.^31^

### Dietary Sources of Trimethylamine N-oxide

The main nutrients used as precursors in the production of TMA in the body are choline and carnitine.^3^ However, in a study conducted in healthy adults, it was found that diet was a weak predictor (0.7%-24.8%) of TMAO variance in body fluids (plasma, urine).^21^

### Choline as a Precursor of Trimethylamine

Choline is an essential nutrient that can be obtained both from food and, to some extent, endogenously produced in the liver. The recommended daily intake of choline is 550 mg for men and 425 mg for women (450 mg for pregnant women and 550 mg for breastfeeding women).^40^ Animal foods such as dairy products, liver, eggs, red meat, poultry, and seafood are the main dietary choline sources. Some plants, such as soybeans, grains, and legumes, also contain choline.^41^

There are water- or fat-soluble forms of choline. Free choline, glycerophosphorylcholine, and phosphocholine are water-soluble forms that enter the portal circulation by transporter-mediated absorption of intestinal choline. The fat-soluble forms are phosphatidylcholine, lysophosphatidylcholine, and sphingomyelin. Phosphatidylcholine can be absorbed by enterocytes after hydrolysis by phospholipase A_2_ to lysophosphatidylcholine. Lysophosphatidylcholine in enterocytes can be re-acetylated to phosphatidylcholine or degraded to glycerophosphorylcholine and then to free choline. Fat-soluble forms of choline enter the lymphatic system and then the portal circulation via chylomicrons, so that they are transmitted to peripheral tissue before the liver.^41,42^ Absorption of free choline from the small intestine occurs via the carrier protein. Choline can reach the large intestine when the choline concentration in the small intestine exceeds the carrier protein saturation.^1,42^ Choline containing a trimethylammonium moiety can be oxidized to betaine and then converted to TMA by betaine reductase enzyme, either directly with the choline TMA lyase enzyme or indirectly via another pathway with the joint activity of the choline dehydrogenase and betaine aldehyde dehydrogenase enzymes.^2^ However, it was reported in a study^43^ conducted with a human colon model that the choline-γ-butyrobetaine-TMA pathway is not active for TMA production from choline in humans, and choline is directly metabolized to TMA by the TMA lyase pathway. A study conducted with single bacterial strains, human fecal microbes, and mouse fecal cell lysates found that the metabolism of choline to TMA could be inhibited by 3,3-dimethyl-1-butanol, which is a choline analogue.^44^ However, this effect of 3,3-dimethyl-1-butanol was not observed in the human colon model.^43^

### The Association Between Choline Intake and Plasma Trimethylamine N-oxide Levels

Although the information on the metabolism of dietary choline to TMA and TMAO has not yet been clarified, it is known that dietary choline intake increases postprandial plasma TMAO levels. The consumption of 2 hard-boiled eggs, each containing about 250 mg of choline, and the intake of 250 mg of labeled phosphatidylcholine supplements have been shown to temporarily double the plasma TMAO levels within 1 hour in healthy individuals.^22^ In another study conducted in healthy individuals, it was found that consumption of 2 or more eggs significantly increased plasma TMAO levels; plasma and urine TMAO levels increased with the number of eggs consumed; plasma TMAO peaked 6-8 hours after egg consumption; and after a meal containing eggs, approximately 14% of the amount of choline taken was converted into TMAO.^45^

Studies examining the effects of longer-term egg consumption support the fact that the effect of egg consumption on plasma TMAO concentration is acute. In a randomized crossover study^46^ conducted in healthy adults of normal body weight, the individuals were divided into 2 groups to consume 2 large eggs or oatmeal (284 g/day) for breakfast for 4 weeks. It was found that plasma choline levels were higher after egg consumption than after oat consumption, but plasma TMAO levels were not significantly different between groups. In another randomized, crossover study,^47^ overweight or obese postmenopausal women with mild hypercholesterolemia were given 2 whole eggs for breakfast for 4 weeks, or an equivalent amount of egg white-only breakfast. It was found that the amount of plasma choline increased significantly after the consumption of whole eggs, but there was no significant difference after the consumption of egg whites. It was reported that plasma TMAO levels were not significantly affected by either application. Moreover, it was reported that the consumption of 3^48^ or 4^26^ eggs per day did not statistically increase the plasma TMAO levels. Although intervention studies have partially indicated that the long-term consumption of dietary choline sources does not cause significant changes in plasma TMAO levels and there is no correlation between diets high in choline and CVD, observational studies do not support these findings. For example, in a study^49^ conducted with healthy men to assess the determinants of plasma TMAO levels, a positive correlation was found between egg consumption and plasma TMAO levels.

The choline source and form are also some of the factors that affect TMAO production from choline. For example, in a study conducted in healthy adults, it was reported that the increase in plasma TMAO levels of individuals taking choline bitartrate supplements was 3 times greater than the group receiving phosphatidylcholine supplements.^27^ Wilcox et al^26^ reported that the effects of 3 different choline sources and forms containing similar amounts of choline (approximately 450 mg) on plasma TMAO levels were not the same. The study found that choline bitartrate supplementation significantly increased plasma TMAO levels and platelet reactivity (*P* < .01 each), but the consumption of 4 large eggs or phosphatidylcholine supplements had no significant effect.

Last of all, it can be inferred that either the choline present in eggs does not have a long-term effect on the plasma TMAO levels or that the amount and duration of egg consumption, as examined in experimental studies, are inadequate to induce notable changes in the TMAO pathway. Furthermore, it is likely that an increase in choline intake will acutely increase plasma TMAO levels and that the source and form of choline, as well as the amount of choline, influence TMAO production.

### Carnitine as a Precursor of Trimethyamine

Meat and dairy products are the main sources of l-carnitine. Its key role is to facilitate the transport of activated long-chain fatty acids to the mitochondrial matrix for oxidation.^50^ It can be synthesized by the human body from the amino acids lysine and methionine.^51^ The compound exerts cardioprotective effects by mitigating inflammation, oxidative stress, and cardiac myocytes’ necrosis. Its regulatory role in endothelial integrity, membrane phospholipid content, intracellular enzyme release, and calcium influx has also been implicated in CVD.^52^

l-carnitine is not absorbed from the small intestine but can be metabolized by bacteria in the large intestine, leading to the production of TMA and malic semialdehyde by breaking the carbon–nitrogen bond in its structure.^53^ TMA can be derived from l-carnitine through direct or indirect pathways. Specifically, l-carnitine can be directly converted to TMA by the carnitine oxidoreductase enzyme, or it can be converted to γ-butyrobetaine and then to TMA by the carnitine TMA lyase enzyme. l-carnitine can be converted bidirectionally into γ-butyrobetaine, which can be produced from carnitine via carnitine-to-carnitine coenzyme A (CoA) transferase or carnitine TMA lyase enzymes. Additionally, l-carnitine can be produced from γ-butyrobetaine through the enzyme γ-butyrobetaine hydroxylase.^2^ However, in the human colon model, no pathway was observed wherein l-carnitine was directly metabolized to TMA by the carnitine oxidoreductase enzyme.^43^

### The Relationship Between Carnitine Intake and Plasma Trimethylamine N-oxide Levels

High doses of carnitine supplements have the potential to increase TMAO concentrations to pathological levels. A systematic review^54^ investigating the health effects of long-term use of l-carnitine supplementation reported that use of 1-4 g l-carnitine per day for 12 or 24 weeks caused an increase in fasting plasma TMAO levels, which may be a risk factor for CVD. In the case of individuals with mitochondrial disorders, long-term (>3 months) supplementation of l-carnitine has been shown to result in an approximately 12-fold increase in the median plasma TMAO levels, rising from 3.54 µm to 43.26 µm after starting daily oral therapy with 1000 mg of l-carnitine.^55^ However, it has been reported that the increases in plasma TMAO levels observed with l-carnitine supplementation were reversed 4 months after cessation of supplementation.^56^ It is important to note, therefore, that the results presented here highlight the risks of high doses of carnitine and the necessity of monitoring TMAO levels in individuals undergoing l-carnitine therapy.

Studies indicate that the consumption of dietary sources of carnitine affects plasma TMAO levels. Consumption of approximately 225 g of beef steak (approximately 180 mg of carnitine) has been found to acutely increase plasma and urinary TMAO concentrations.^19^ In a three-arm, randomized, cross-controlled trial,^57^ red meat consumption was found to increase plasma and urinary TMAO levels in healthy individuals, but consumption of white meat or non-meat alternatives (plant sources and milk) did not make a significant difference to TMAO. On average, red meat consumption was associated with a threefold increase in plasma TMAO levels compared with white meat and non-meat alternatives. However, the findings of cross-sectional studies evaluating the relationship between dietary meat intake and plasma TMAO concentrations are conflicting. Some studies indicate that there is no noteworthy association between the consumption of red, white, or processed meat and plasma TMAO concentrations,^49,58^ yet a study has also demonstrated that individuals with plasma TMAO levels higher than 4.60 µmol/L consume more red meat in their diets.^23^ A critical analysis has highlighted that to increase TMAO to pathological levels (>10 μM) in the long term, high and chronic dietary levels of carnitine are required.^3^

In summary, both l-carnitine supplementation and consumption of red meat, a dietary source of l-carnitine, are associated with an increase in plasma TMAO levels. However, a similar effect cannot be inferred for other dietary sources of l-carnitine (white meat, dairy products) from the available data.

### Direct Dietary Source of Trimethylamine N-oxide: Seafood

Cellular adaptation to changes in osmotic and hydrostatic pressure and regulation of cell volume are mediated by TMAO. It is therefore naturally present in deep-sea species in particular.^1,58^ Furthermore, the concentration of TMA and TMAO in fish and other seafood varies depending on the species and habitat. For example, combined TMA and TMAO concentrations are significantly lower in clams, wallace, and other freshwater fish, as well as canned tuna, but significantly higher in some deep-sea fish such as orange roughy, cod, and halibut. It should be noted that farmed salmon had lower total TMA + TMAO levels than wild salmon.^59^

Trimethylamine N-oxide can be absorbed directly from the intestine within 15 minutes following seafood consumption, without the need for gut microbiota fermentation and hepatic processing.^24,60^ Additionally, the consumption of fish increased postprandial plasma TMAO levels significantly more than the intake of eggs or beef, by approximately 50 times.^24^ Several cross-sectional studies have also consistently associated higher fish consumption with higher plasma^21,23,49^ and urinary^21,61,62^ TMAO concentrations. Considering the positive correlations between consuming seafood and plasma TMAO concentrations, along with evidence suggesting a reduced risk of CVD with fish consumption,^63,64^ it can be inferred that there may be an inconsistency in the association between seafood consumption and CVD. To better understand the relationship between CVD, TMAO concentration, and seafood consumption, studies are needed that examine seafood consumption in more detail, such as the type of fish consumed.

As a preventive strategy to reduce the risk of CVD events, it may be wise to choose fish with low total TMA + TMAO and high EPA and DHA levels (such as trout, farmed salmon, and white seabass) when eating fish, especially for risk groups such as those with CVD disease or chronic kidney failure.

### Microbiota Related to Plasma Trimethylamine N-oxide Levels

The role of microbiota in preventing and treating noncommunicable diseases is increasing by the day. The microbiota is considered a prominent link between CVD and modifiable risk factors.^65^ When considering the evidence that the gut microbiota produces TMA,^6^ which is then converted into TMAO, and the observed links between increased plasma TMAO levels and adverse cardiovascular events,^10-18^ the conclusion that the microbiota plays a mediating role in linking diet and CVD is strengthened.

Circulating levels of TMAO are influenced by variations in the ability of the gut microbiota to produce TMA. The absence of TMA in the urine of germ-free mice without microbiota,^66^ the suppression of TMAO production from dietary choline^22^ and carnitine^19^ in mice treated with broad-spectrum antibiotics, and the re-increase in plasma TMAO levels after antibiotic withdrawal clearly indicate the role of gut microbiota in TMA and TMAO production. A study^19^ on omnivores and vegetarians revealed that, despite both groups being provided with an equivalent amount of l-carnitine supplementation, the increase in plasma and urinary TMAO levels was significantly higher in omnivores than in vegetarians. This study highlighted the crucial role of the established gut microbiota.

Mainly, the Firmicutes and Proteobacteria phyla, as well as *Anaerococcus hydrogenalis*,* Escherichia fergusonii*,* Clostridium asparagiforme*,* Clostridium hathewayi*,* Providencia rettgeri*, *Clostridium sporogenes*,* Proteus penneri*, and *Edwardsiella tarda*, play a role in the breaking of the carbon–nitrogen bond in the choline structure and the formation of TMA.^67^ The degradation of the 3-hydroperoxybutyryl moiety of l-carnitine and the subsequent formation of TMA are attributed to *Proteobacteria* and *Bacteroidetes* at the phylum level and *Prevotellaceae* at the family level, based on reports.^7,19^ In addition, plasma TMAO levels were found to be higher in individuals with a *Prevotella* dominant enterotype compared to those with a *Bacteroides* dominant enterotype.^19^ In parallel with this, Fennema et al’s^7^ review indicates that various families of bacteria can produce TMA, yet they are more prevalent within the *Firmicutes* phylum and comparatively less common within the *Bacteroidetes* phylum. A cross-sectional analysis of data from the Multi-Ethnic Cohort Study involving 1653 participants aged 60-77 years reported that plasma TMAO levels were associated with the abundance of 13 genera, including 6 Firmicutes, 3 Protobacteria, 3 Bacteroidetes, and 1 Fusobacterium.^23^ Furthermore, research dividing participants into low or high TMAO producers according to their response to TMAO precursors as supplements^27^ or dietary sources^24^ also supported that the gut microbiota differed between groups and that high TMAO producers had more Firmicutes than Bacteroidetes.

While research into this topic is currently limited, current studies indicate that the microbiota has a decisive role in the synthesis of TMAO from dietary choline and carnitine. Additional research is required to identify correlations between plasma TMAO levels and particular genera. In the future, researchers may conduct studies to evaluate the impact of modifying the gut microbiota through dietary intervention or supplementation on plasma TMAO levels. In the future, personalized dietary advice with a focus on the gut microbiome may be considered as a potential strategy to decrease plasma TMAO levels and mitigate the risk of CVD.

## Conclusion

There is compelling evidence of a link between plasma TMAO levels and CVD. Nevertheless, the mechanisms contributing to the pathological rise in plasma TMAO levels remain inadequately comprehended. Based on the scientific evidence available, consumption of dietary sources containing TMAO precursors, such as choline and carnitine, results in an acute increase in circulating TMAO levels. This increase is even more pronounced with dietary supplements, whereas the effect of long-term consumption of TMAO precursors, reflecting dietary habits, is less clear. However, it is well documented that the intestinal microbiota plays a decisive role in the relationship between diet and plasma TMAO levels. Furthermore, there is a strong relationship between consumption of seafood, which is a direct dietary source of TMAO, and plasma TMAO levels. For these reasons, it may be advisable to avoid choline and carnitine supplements unless they are needed and, if necessary, to monitor plasma TMAO levels regularly and to consume fish with low levels of total TMA and TMAO, especially for those at risk of CVD.
